# Cases Admitted under the Care of the Surgeon of the Finsbury Dispensary, St. John's Square, Clerkenwell, from the 10th of August to the 10th of September, 1802

**Published:** 1802-10-01

**Authors:** J. Ricards

**Affiliations:** No. 68, Hatton Garden


*Cases admitted under the Care of the Surgeon of the Finsbury Dispensary, St. John's Square, Clerkenwell, from the* 10*th of Auguſt to the* 10*th of September*, 1802.

| | | | |
|---|---|---|---|
| Phlegmone Artuum - - | 2 | Contuſiones - - - - - - | 6 |
| ——— Teſtis - - - | 1 | Lues - - - - - - - - | 3 |
| ——— Oſſis Sterni - - | 1 | Gonorrhœa - - - - - - | 4 |
| Eryſipelas - - - - - - | 2 | Scrophula - - - - - - | 2 |
| Abſceſſus Artuum - - - | 4 | Herpes - - - - - - - | 1 |
| ——— Articulorum - | 3 | Tinea - - - - - - - - | 2 |
| ——— Mammæ - - - | 3 | Pſoriaſis - - - - - - - | 1 |
| Mortificatio Artûs - - - | 1 | Eruptio Papuloſa - - - | 1 |
| Ulcera Faucium - - - - | 2 | Tumor Albus Carpi - - | 1 |
| ——— Artuum - - - - | 8 | Prolapſus Uteri - - - - | 1 |
| Fiſtula in Ano - - - - | 1 | Clavus - - - - - - - | 1 |
| Leucoma - - - - - - | 2 | Paralyſis Artuum - - - | 3 |
| Fractura Coſtarum - - - | 2 | Hæmaturia - - - - - - | 1 |
| Luxatio Carpi - - - - | 1 | | |
| Vulnera Capitis - - - - | 2 | Total | 66 |
| ——— - Artuum - - - | 3 | | |

It is an obſervation which is daily made by Surgeons, of which experience ſufficiently proves the validity, that the diſeaſes of no organs which fall under their notice, preſent to them ſo great difficulty in the management, as diſeaſes of the bones. We ſhould, indeed, à priori, without the aſſiſtance of practical obſervation, have ſuppoſed this to be the caſe, from an examination of the peculiar ſtructure of theſe organs; for we find that they are equally vaſcular, and as liable to diſeaſe as ſofter parts; with this particular diſadvantage, that their vaſcular ſtructure is enveloped in a matrix, which preſents difficulties to the evolution and production of thoſe phenomena which are are characteriſtic of diſeaſe; and therefore, in its progreſs, more exertion is neceſſary, and more injury ſuſtained, in the proper accompliſhment of thoſe alterations of ſtructure or of form.

If a bone inflames, as inflammation cannot exiſt without ſome degree of ſwelling; and as the bones are firm and unyielding, as the oſſeous matter, while in its healthy ſituation, will not ſuffer the veſſels to enlarge, it becomes a point of neceſſity, that a part of the earth of the bone ſhould be removed to a greater diſtance from the expanding veſſels; which is the fact. If an abſceſs forms in the bone, a proportionate opening muſt be made by the abſorbents, through the bony fabrick, to give egreſs to the matter; which being a work of difficulty, the cavity of the bone ſometimes becomes filled with matter, and the moſt dreadful diſeaſe is the conſequence. If a part mortifies, a ſeparation muſt take place between the ſound and unſound parts. Should the dead portion, happily, be ſituate on the external part of the bone, it is more eaſily caſt off; but ſhould the internal part be carious from deſtruction of the medullary artery, a difficulty occurs which Nature can ſeldom overcome, and the limb generally falls a ſacrifice to the diſeaſe.

The powers of Nature, therefore, in diſeaſes of the bones, exert themſelves to accompliſh the ſame means of redreſs, as in analogous diſeaſes of other parts; but the peculiar ſtructure of theſe organs not unfrequently renders theſe efforts inefficient.

I have been led to reflections on theſe diſeaſes by ſeveral circumſtantial facts which have recently preſented themſelves to my obſervation; which have inclined me to believe that bones feel the effects of ſlight external cauſes oftener than is generally imagined.

The patient in the above liſt, (Phlegmone Sterni) above four months ago, received an accidental blow on the ſternum, by the elbow of her huſband while aſleep, which was not regarded at the time, nevertheleſs ſhe felt ſome degree of pain in the part, at times, of a dull obtuſe kind, which however, did not excite in her mind any alarm, till within a month, (three months after the accident) when ſhe thought that there was ſome enlargement, and the pain increaſed to ſuch a degree, that a week ago ſhe preſented herſelf for advice and aſſiſtance; there is now very conſiderable enlargement of the ſternum, evidently of the bone, and ariſing either from an enlargement of the ſubſtance of the bone itſelf from the tumefaction which always takes place in inflammation, or from the formation of an abſceſs in the anterior cavity of the mediaſtinum, which, by its diſtenſion, propells the bone forward. The patient, as is generally the caſe in diſeaſes of the bones, is conſtitutionally affected, extremely debilitated, with an entire loſs of appetite,

ſleepleſs

ſleepleſs nights, and conſiderable pain. The ſmall length of time in which ſhe has been uſing the means for recovery, will not allow of any ſatisfactory information reſpecting the progreſs of this diſeaſe; the pain, however, is very conſiderably abated by the effects of one bliſtering plaſter which has been applied. It is my intention to continue the uſe of theſe, applied in ſucceſſion as faſt as cicatrization ſhall take place, with the internal exhibition of cinchona, opium, &c. &c.

Mr. Hill, of Cheſter, in a late Number of the Medical and Phyſical Journal (vol. vii. p. 415) has made ſome very judicious remarks on diſeaſes of the bones, and has given caſes of the kind wherein bruiſes of the bones have produced very formidable diſeaſes, and death, from a depoſition of bony matter on the external ſurface of the bone; and he ſays "Perhaps no hurt, however ſmall, is inflicted on a bone without a greater or leſs degree of this proceſs taking place." I know that in caſes wherein bruiſes have been inflicted on a part where the bone lies very near the ſurface, being covered with little more than integuments, the effects of that bruiſe are ſenſible to us much longer than would be the caſe in parts of a ſofter nature.

A gentleman received a kick from a horſe on the chin, which did not abrade the cuticle, yet he experienced a pain upon preſſure on that ſpot many years, and the bone ſeemed to the touch enlarged on that ſide. Another gentleman ſuſtained a blow on the ſhin, which juſt grazed the external integuments, and a ſlight degree of enlargement of the part with pain on preſſure, has remained ever after. A lady, by a fall from a horſe, ſcratched her face by the gravel of the road, and ever ſince has experienced conſiderable tenderneſs of the noſe (the bone being at that part moſt thinly covered) from the friction of a towel. Do theſe trivial effects, which remain after ſlight accidents of this kind, proceed from oſſific inflammation and depoſition, or from perioſteal thickening only?

We cannot, in diſeaſes of the bones, give that decided relief which we are enabled to adminiſter to ſimilar diſeaſes of ſoft parts, nevertheleſs we are able to afford very conſiderable aſſiſtance; it is promulgated, as an idea, by ſome, that we ſhould not interfere in the ſeparation of carious bone, as Nature is fully competent to the performance of this operation. Nature and time certainly will accompliſh the exfoliation of portions of bone of any magnitude, if on the external part of the bone, provided the conſtitution will ſupport the effects of the means which ſhe employs: for as a portion of carious bone muſt be conſidered as a body extraneous to the ſyſtem; a body not entering into the channel of the circulation; an extraneous body of of the moſt miſchievous kind; as, while this remains, there will exiſt ulceration of the ſuperincumbent ſoft parts, perpetual diſcharge, continual irritation, and frequently re-iterated attacks of inflammation, if the extent of the diſeaſe be conſiderable, the vigor of the moſt robuſt habit muſt decline; I would therefore always, where, from the local ſituation of the diſeaſe, the operation were practicable, prefer the more active practice of removing the diſeaſed portion, to the more paſſive, tedious, and often hazardous cuſtom of committing the caſe to Nature. I have a caſe at preſent under my care, of a man, who for a long period of time (the exact date eſcapes my recollection) has had a diſeaſe in the bones of the ſternum; he has ſeveral ſmall apertures through the integuments and muſcles, from which exudes a continual purulent diſcharge; and through which ſinuſes extend to the bones, which may be diſtinctly felt by the probe, in a carious ſtate; he has very frequent attacks of inflammation of the adjacent parts, which are attended with extreme pain, and general irritation and diſtreſs. I have recommended, and certainly would perform, (were the patient willing) an operation for the removal of the offending cauſe, which might be done with perfect facility by expoſing the bone and ſeparating the morbid portions by a ſmall trephine.

In fact, I think that the removal of extraneous matter from the body, whether of dead portions of bone, formed within the body, or of other ſubſtances which may in any way have gained acceſs, ought to be the invariable *endeavour* of ſurgeons; there may be caſes where an exception may be made to this as a general axiom, where perhaps the continuance of the extraneous body may do leſs injury than an operation for its extraction; where the body is of an obtuſe, ſmooth, and globular figure, as bullets, &c. which have frequently remained many years buried in the body, forming for themſelves a ſac, without producing ill effects, if unaccompanied by wadding, pieces of cloth, &c. But from the neglect of, or the unſucceſsful attempts in removing extraneous bodies of a different form, I have been witneſs to the moſt dreadful effects. A caſe of this kind has recently occurred to me, which terminated fatally, from an apparently trivial cauſe. The patient by a fall from a chair on which he was ſtanding, received a wound juſt over the great trochanter of the thigh, by a piece of the birch of a broom on which he fell, which he immediately drew from the wound, which was apparently of no conſequence, as it ſeemed merely to penetrate the ſkin; he accordingly did not notice it, but was in more pain than he could have expected from ſuch a circumſtance. In a week my attendance was deſired, when I found found a confiderable degree of tenfion and inflammation of the *anterior part of the thigh*, and about the *groin*, but the integuments about the *wound* were flaccid, and the *wound* itfelf nearly *healed*; I feared much that fome portion of the wood remained in the thigh, though he repeatedly affured me that it could not be, from the nature of the piece which he extracted, it being fmoothly cut at the end and not ragged, as it would have been were it broken in the wound. Being however rather fceptical refpecting his affertions, I enlarged the orifice to fome extent, but could difcover no remnant of the wounding fubftance; indeed, the fwelling was far from the orifice, and continued to recede farther from it, and in the courfe of a fortnight an abfcefs pointed diametrically oppofite to the trochanter; this abcefs being opened, it continued to difcharge moft profufely for feveral weeks, during which time the patient became moft completely hectic, in refiftance to all the fupport which was contributed to his relief, when a thick piece of wood, about half an inch in length, was difcharged; fo much exhauftion had however previoufly taken place, that he died in a few days. It may reafonably be inferred, that had chirurgical affiftance been procured in the firft inftance, before the offending caufe had eluded obfervation, no ill confequences would have occurred; but at the time I firft faw him, as the moft prominent part of the fwelling was over the groin and upper part of the thigh, extending inwards, it is probable that the offending caufe was in that fituation, paffing over the bone, and under the large veffels of the thigh. It is fomewhat remarkable that the original wound fhould heal with fuch a body in it, as it was nearly cicatrized before I enlarged it, nor was there any finus fhewing the direction of the wound, the parts healing behind the extraneous body as it paffed along; and afterwards it again healed as readily and with as little difcharge as a fimple wound of the fame extent made by the knife could have done; this circumftance induced me to believe that I had in the firft inftance entertained an erroneous idea refpecting the exiftence of fome portion of the wood in the limb, till I was ocularly convinced of it by its appearance in the abfcefs.

*No. 68, Hatton Garden.* J. RICARDS.

*A Reply*

